# A nomogram for predicting the in-hospital mortality after large hemispheric infarction

**DOI:** 10.1186/s12883-019-1571-4

**Published:** 2019-12-29

**Authors:** Wenzhe Sun, Guo Li, Ziqiang Liu, Jinfeng Miao, Zhaoxia Yang, Qiao Zhou, Run Liu, Suiqiang Zhu, Zhou Zhu

**Affiliations:** 1 Department of Neurology, Tongji Hospital, Tongji Medical College, Huazhong University of Science and Technology, No.1095 Jiefang Avenue, Wuhan, 430030 China; 2 Department of Radiology, Tongji Hospital, Tongji Medical College, Huazhong University of Science and Technology, No.1095 Jiefang Avenue, Wuhan, 430030 China; 3 Department of Geriatrics, Taikang Tongji Hospital, No.233 SiXin North Road, Wuhan, 430030 China

**Keywords:** Large hemispheric infarction, Neutrophil-to-lymphocyte ratio, In-hospital mortality, Inflammation, Nomogram

## Abstract

**Background:**

Large hemispheric infarction (LHI) is a severe form of stroke with high mortality and disability rates. The purpose of this study was to explore predictive indicators of the in-hospital mortality of LHI patients treated conservatively without decompressive hemicraniectomy.

**Method:**

We performed a retrospective study of 187 consecutive patients with LHI between January 1, 2016 to May 31, 2019. The receiver operating curves were preformed to evaluate predictive performance of demographics factors, biomarkers and radiologic characteristics. Significant prognostic factors were combined to build a nomogram to predict the risk of in-hospital death of individual patients.

**Result:**

One hundred fifty-eight patients with LHI were finally enrolled, 58 of which died. Through multivariate logistic regression analysis, we identified that independent prognostic factors for in-hospital death were age (adjusted odds ratio [aOR] = 1.066; 95% confidence interval [CI], 1.025–1.108; *P* = 0.001), midline shift (MLS, aOR = 1.330, 95% CI, 1.177–1.503; *P* <  0.001), and neutrophil-to-lymphocyte ratio (NLR, aOR = 3.319, 95% CI, 1.542–7.144; *P* = 0.002). NLR may serve as a better predictor than white blood count (WBC) and neutrophil counts. Lastly, we used all of the clinical characteristics to establish a nomogram for predicting the prognosis, area under the curve (AUC) of this nomogram was 0.858 (95% CI, 0.794–0.908).

**Conclusion:**

This study shows that age, MLS, and admission NLR value are independent predictors of in-hospital mortality in patients with LHI. Moreover, nomogram, serve as a precise and convenient tool for the prognosis of LHI patients.

## Background

Large hemispheric infarction, also known as malignant middle cerebral artery infarction (MMI) [1], is one of the most malignant types of supratentorial ischemic stroke. It has an annual incidence of 10~20 per 100,000 people and a mortality rate of 30%~ 80% [1–4]. It is used to describe total or partial anterior circulation infarct caused by occlusion of internal carotid artery or the proximal middle cerebral artery (MCA), affecting a large portion of the MCA territory and constituting up to 10% of all supratentorial ischemic strokes [5]. To date, effective conservative treatment of LHI remains unsolved. Three European trials, DESTINY, DECIMAL, HAMLET and a recent study DESTINY II, all demonstrate that decompressive hemicraniectomy (DHC) improves survival and outcome [6, 7]. However, only 0.3% of all ischemic stroke patients would be eligible for DHC according to the strict eligibility criteria in the European hemicraniectomy trials [8]. Meanwhile, the willingness of family members and patients also decreases the rate of DHC, since reduction in mortality with DHC was accompanied by an increase in moderate severe disability in survivors. Therefore, identification of early mortality factors and survival factors for LHI patients who do not receive DHC remains important for clinical decision making.

Factors affecting the mortality and prognosis of LHI are not fully understood. Although malignant brain edema is usually considered as the leading cause of early death of LHI [1], it is noted that not all patients with LHI would develop the malignant cerebral edema. In addition, apart from cerebral edema, many other factors such as hemorrhagic complications and poststroke infections can also lead to patients’ death [9, 10]. Therefore, further studies for identifying the predictors, especially accurate and measurable prediction model for prognosis, are pivotal for risk-optimized therapeutic strategies and allocation of healthcare resources. The objective of this retrospective study was 1) to investigate the predictors associated with in-hospital mortality in patients with LHI. 2) to establish a comprehensive visual predictive nomogram of in-hospital mortality in LHI patients, calculating a probabilistic estimate for clinicians.

## Methods

### Study cohort

**Fig. 1:**
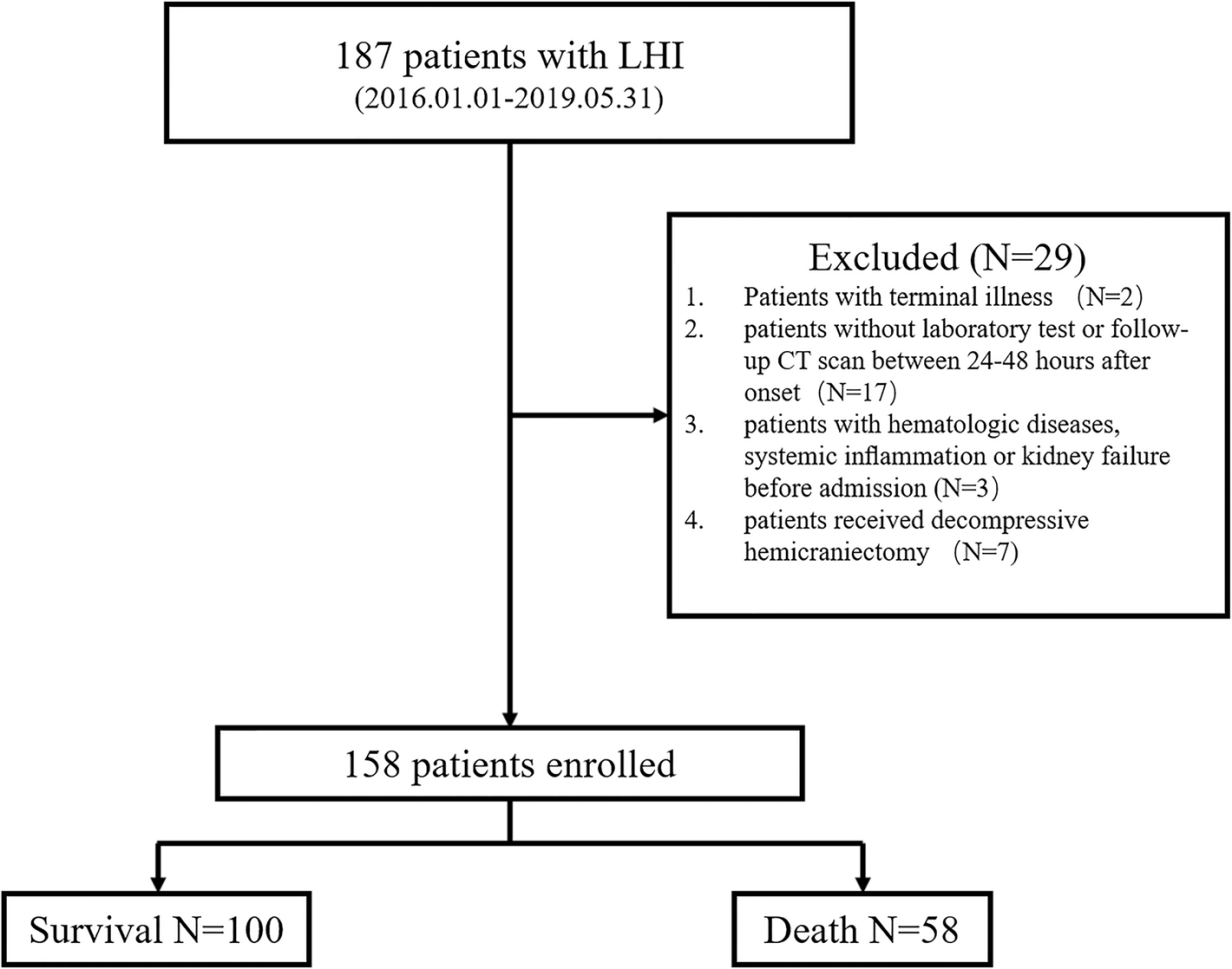
Enrollment flow chart of study cohort

This study retrospectively recruited 187 adult patients diagnosed with LHI and admitted within 48 h to the neuro-intensive care unit (NICU) of Tongji Hospital at Huazhong University of Science and Technology between January 2016 and May 2019. This retrospective study was approved by our Institutional Review Board of Tongji hospital and written informed consent was waived. The enrollment flow chart is shown in Fig. 1.

### Inclusion and exclusion criteria

LHI was defined as infarction involving at least 50% of the territory of the middle cerebral artery (MCA) in computed tomography (CT) scan and/or diffusion weighted image (DWI), with an acute onset of corresponding clinical signs and symptoms [11, 12]. All patients completed the baseline CT scan or DWI at admission and had no primary intracranial hemorrhage. Other inclusion criteria for our study were: 1) Chinese ethnicity; 2) age 18 years or older; 3) first or recurrent acute stroke occurring within 48 h before admission. The exclusion criteria included: 1) Patients with terminal illness such as tumor, severe trauma, or other life-threatening diseases before admission; 2) patients with hematologic diseases, systemic inflammation or kidney failure before admission; 3) patients without laboratory test or follow-up CT scan, the follow-up CT was used to determine if there is a hemorrhagic transformation; 4) patients who received decompressive hemicraniectomy. Hemorrhagic transformation during hospitalization was not an exclusion criterion and ECASS II classification was used to determine its severity.

### Imaging assessment and data collection

The demographic characteristics were collected consisting of age, gender, history of smoke and history of alcohol intake, baseline temperature, baseline systolic pressure and baseline diastolic pressure. The following laboratory tests at admission within 48 h of syndrome onset were collected: baseline blood glucose, HbA1c, neutrophil counts, lymphocyte counts, high-sensitivity C-reactive protein, triglyceride (TG), high-density lipoprotein (HDL), low-density lipoprotein (LDL), NLR was calculated by dividing the neutrophil counts by the lymphocyte counts. In-hospital treatment analyzed in this cohort included acute intervention (thrombolysis or thrombectomy). We retrospectively studied all 158 patients’ radiology data on CT scan undergone between 24 h and 48 h after syndrome onset. MLS is defined as the distance from the septum pellucida to the anatomic line anchored by the falx cerebri to the skull [13]. All radiology images were assessed and measured by an experienced radiologist, blinded to the patients’ outcome. Most patients didn’t have National Institutes of Health Stroke Scale score (NIHSS), except the ones who received acute interventional therapy. Thus, NIHSS was not included as a variable.

### Statistical analysis

Statistical analyses were performed using R version 3.5.2 software (Institute for Statistics and Mathematics, Vienna, Austria; http://www.r-project.org/), IBM SPSS statistics 22 software (SPSS Inc., Chicago, IL, United States) and MedCalc v15.8 (MedCalc Software, Ostend, Belgium). The total percentage of missing values was 1.41. Missing values were replaced through multiple imputation to reduce bias and increase statistical power [14]. The imputation technique involves creating multiple copies of the data and replacing missing values with imputed values through a suitable random sample from their predicted distribution. We used the “mice” package of the statistical package R (version 3.5.2) to obtain 5 imputation datasets.

Continuous variables (including MLS) are presented with their medians and interquartile ranges (IQR). Categorical variables are presented as numbers (percentages). We transformed NLR values and Hypersensitive C-reactive protein (hs-CRP) into a log scale. Univariate analyses were conducted using univariate logistic regression analysis. Variables with *P* <  0.10 from the results of the univariate analyses were considered confounders in the multivariable logistic regression analysis. All *P*-values were two-sided, and *P* <  0.05 was considered statistically significant. In order to compare NLR and conventional inflammation, a non-parametric statistical analysis of differences between areas under correlated curves was performed, according to DeLong et al. [15]. The “rms” package (cran.r-project. Org/web/packages/ rms) was used to construct nomogram models.

## Results

### Patient characteristics

A total of 106 men and 52 women were retrospectively recruited into this cohort. The median age was 61 years (IQR 53~71). Among these patients, 38 (24.1%) received acute interventional treatment (Thrombolysis or thrombectomy), 102 patients (64.6%) completed magnetic resonance imaging (MRI), 19 (12.0%) patients developed hemorrhagic transformation and 58 (36.7%) subjects died during hospitalization (median survival time was 7d [IQR 4~14.5]). In this cohort, the median length of stay (LOS) in dead patients is 5 (IQR, 3~11) and surviving patients is 16 (IQR, 11~21). The reasons of in-hospital death in this cohort included brain herniation in 26 (44.8%) patients, neurologic decline without brain herniation in 3 (5.2%), respiratory failure in 13 (22.4%), sepsis in 6 (10.3%), cardiac arrest in 7 (12.1%) and multi-organ failure in 3 (5.2%) patients. All surviving patients withdraw intensive care, 83 patients go to the rehabilitation department for further treatment and 17 patients choose to go home after discharge. Among the patients who survived discharge, the modified Rankin scale (mRS) score was 2 in 18 patients,3 in 20 patients, 4 in 54 patients, 5 in 8 patients, and the median mRS was 4. Further baseline patient characteristics are presented in Table 1.

**Table 1:** Patient demographics, laboratory information and radiologic characteristics (*n* = 158)

parameter	All (*N* = 158)	Survival (*N* = 100)	Death (*N* = 58)
Age (years)	61 (53~71)	58 (50~68)	68 (60~75)
Female (%)	52 (32.9)	31 (31)	21 (36.2)
Smoke (%)	80 (50.6)	51 (51)	29 (50)
Alcohol (%)	63 (39.9)	43 (43)	20 (34.5)
ECASS-II>2 (%)	13 (8.2)	5 (5)	8 (13.8)
Acute Intervention (%)	38 (24.1)	23 (23)	15 (25.9)
MLS (mm)	2.5 (0~5.9)	0 (0~4.0)	6.0 (1.4~10.5)
Baseline temperature (°C)	36.5 (36.4~36.8)	36.5 (36.3~36.8)	36.5 (36.5~37)
SBP (mmHg)	145 (126~165)	146 (126~166)	145 (126~159)
DBP (mmHg)	82 (74~93)	83 (72~95)	80 (74~91)
GLU (mmol/L)	6.2 (5.5~7.6)	5.9 (5.4~6.8)	7.1 (5.9~9.2)
HbA1c (%)	5.7 (5.3~6.2)	5.6 (5.3~6.1)	5.8 (5.3~6.4)
hs-CRP (mg/L)	11.1 (3.4~30.2)	8.4 (2.6~20.9)	15.9 (5.2~45.6)
NLR	5.9 (4.3~10.2)	5.2 (3.8~7.8)	8.6 (5.4~14.6)
TG (mmol/L)	1.1 (0.8~1.52)	1.0 (0.7~1.6)	1.2 (0.9~1.4)
HDL (mmol/L)	1.2 (0.9~1.4)	1.2 (0.9~1.4)	1.2 (1.0~1.3)
LDL (mmol/L)	2.7 (2.2~3.3)	2.6 (2.2~3.3)	2.7 (2.2~3.3)

*ECASS-II* European Cooperative Acute Stroke Study-II classification, *MLS* midline shift, *SBP* baseline systolic pressure, *DBP* baseline diastolic pressure, *GLU* baseline blood glucose, *HbA1c* glycated hemoglobin, *hs-CRP* high sensitivity C-reactive protein, *NLR* Neutrophil to lymphocyte ratio, *TG* Triglyceride, *HDL* high-density lipoprotein, *LDL* low density lipoprotein

### Independent predictors for in-hospital death

Logistic regression analysis was performed to explore the factors were associated with in-hospital death. Table 1 shows demographics, laboratory information and imaging material of patients with LHI.

We included MLS as a continuous variable into the logistic regression model for analysis, NLR and hs-CRP were transformed by a log scale for analysis. As shown in Table 2, age, MLS, ECASS-II classification, baseline blood glucose, _log_NLR, _log_hs-CRP were significantly correlated with in-hospital death in univariate logistic regression analyses (P < 0.1). A multivariate logistic regression analysis was used to further explore the contribution of all variables that were shown to be significant in the univariate analysis. In multivariate logistic regression analysis (Table 3), the age (adjusted odds ratio [aOR] = 1.066; 95% confidence interval [CI], 1.025–1.108; *P* = 0.001, MLS (aOR = 1.330, 95% CI, 1.177–1.503; *P* < 0.001) and _log_NLR (aOR = 3.319, 95% CI, 1.542–7.144; *P* = 0.002) remained significant (*p* < 0.05) after adjusting for confounders.

**Table 2:** Univariate logistic regression analysis for in-hospital mortality

parameter	β	SE	*P*	OR (95% CI)
Age	0.061	0.015	< 0.001*	1.063 (1.033~1.095)
Gender (Female)	0.234	0.348	0.502	1.263 (0.638~2.501)
Smoke	− 0.040	0.330	0.904	0.961 (0.503~1.835)
Alcohol	−0.360	0.342	0.293	0.698 (0.357~1.364)
ECASS-II>2	1.112	0.596	0.062*	3.040 (0.945~9.782)
Acute Intervention	0.115	0.383	0.685	1.168 (0.457~2.484)
MLS	0.252	0.055	<0.001*	1.287 (1.155~1.434)
Baseline temperature	0.315	0.321	0.326	1.370 (0.731~2.570)
SBP	−0.006	0.007	0.380	0.994 (0.982~1.007)
DBP	−0.006	0.010	0.568	0.994 (0.974~1.014)
GLU	0.272	0.078	0.001*	1.312 (1.126~1.529)
HbA1c	0.033	0.108	0.763	1.033 (0.836~1.277)
hs-CRP^a^	0.298	0.113	0.008*	1.347 (1.080~1.680)
NLR^a^	1.311	0.310	< 0.001*	3.710 (2.020~6.814)
TG	−0.157	0.230	0.494	0.854 (0.544~1.341)
HDL	−0.329	0.510	0.518	0.719 (0.265~1.955)
LDL	−0.046	0.183	0.802	0.955 (0.667~1.368)

*Variables with *P* < 0.10 in univariate analysis were included in multivariable logistic regression models for adjustment

^a^These variables were transformed into log scale.

*ECASS-II* European Cooperative Acute Stroke Study-II classification, *MLS* midline shift, *SBP* baseline systolic pressure, *DBP* baseline diastolic pressure, *GLU* baseline blood glucose, *HbA1c* glycated hemoglobin, *hs-CRP* high sensitivity C-reactive protein, *NLR* neutrophil to lymphocyte ratio, *TG* Triglyceride, *HDL* high-density lipoprotein, *LDL* low density lipoprotein, *OR* odds ratio, *CI* confidence interval, *SE* standard error

**Table 3:** Multivariate logistic regression model for in-hospital mortality

parameter	β	SE	*P*	OR (95% CI)
Age	0.064	0.020	0.001*	1.066 (1.025~1.108)
ECASS-II > 2	−0.445	0.922	0.630	0.641 (0.105~3.908)
MLS	0.285	0.062	<0.001*	1.330(1.177~1.503)
GLU	0.132	0.085	0.121	1.141 (0.966~1.349)
hs-CRP^a^	0.129	0.151	0.393	1.138 (0.846~1.531)
NLR^a^	1.200	0.391	0.002*	3.319 (1.542~7.144)

*Statistically significant at p < 0.05 level, two-sided;

^a^These variables were transformed into log scale.

*ECASS-II* European Cooperative Acute Stroke Study-II classification, *MLS* midline shift, *GLU* baseline blood glucose, *hs-CRP* high sensitivity C-reactive protein, *NLR* neutrophil to lymphocyte ratio, *OR* Odds Ratio, *CI* confidence Interval, *SE* standard error

**Fig. 2:**
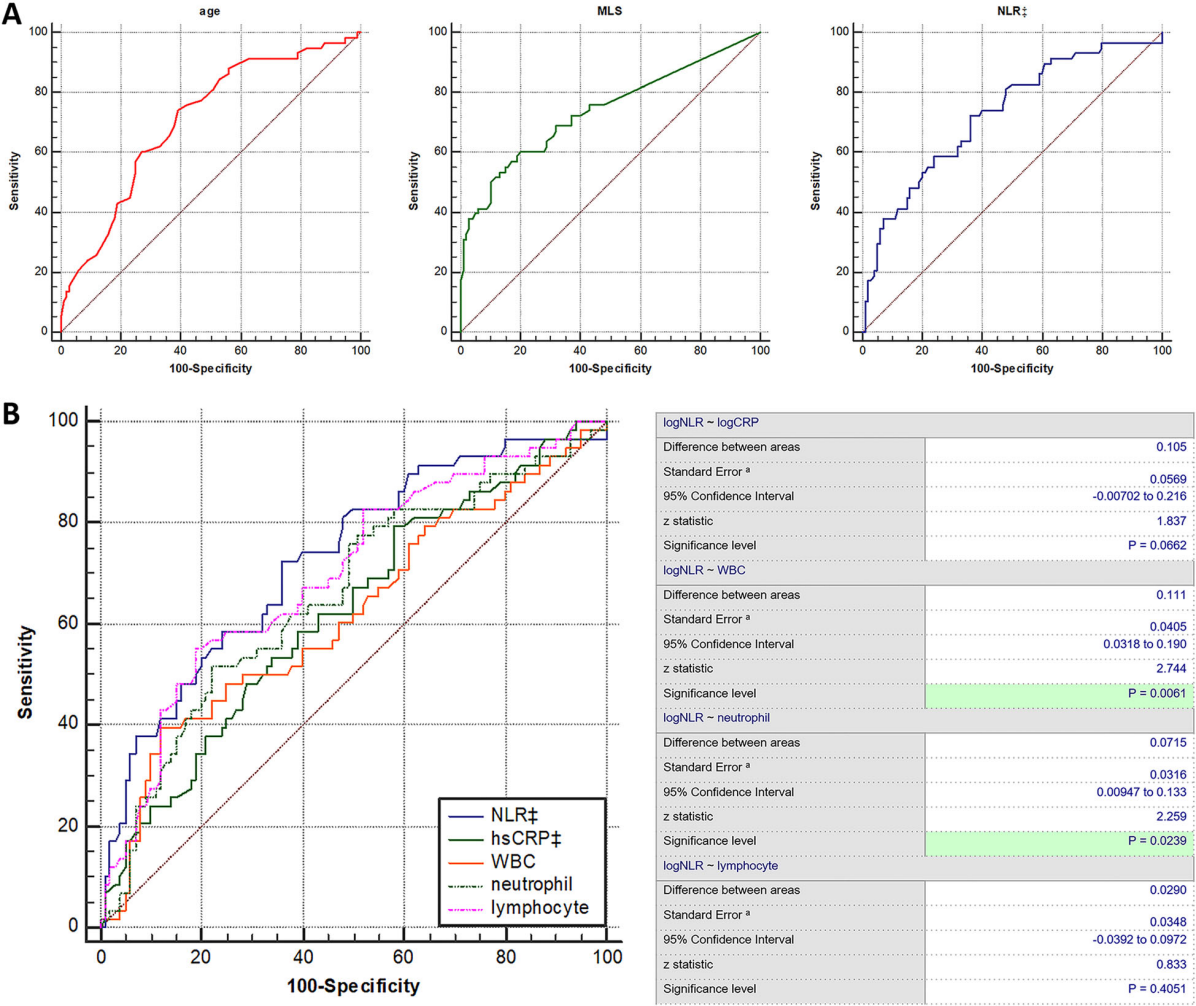
**a**. Discriminative ability presented as receiver operating curves of predictors; **b** Comparison of predictive power between NLR and conventional inflammatory markers in the prediction of in-hospital death. MLS: midline shift; NLR: Neutrophil to lymphocyte ratio; hs-CRP: high sensitivity C-reactive protein, ‡These variables were transformed into log scale

The optimal cut-off values for the independent predictors were calculated by applying a receiver operating curve analysis to test all possible cutoffs that would discriminate between death and survival (Fig. 2a). The area under the curve (AUC) for the ability of age, MLS and _log_NLR at admission to predict in- hospital death were 0.707 (95% CI, [0.630~0.777], optimal cutoff age = 60y, 74.1% sensitivity and 61.0% specificity), 0.738 (95% CI, [0.663~0.805], optimal cutoff values = 5.4 mm, 53.4% sensitivity and 87.0% specificity) and 0.728 (95% CI, [0.651~0.795], optimal cutoff value = 1.78, 72.4% sensitivity and 64.0% specificity), respectively. In comparing the predictive power between NLR and conventional inflammatory markers, _log_NLR (0.728 [0.651~0.795]) showed a higher AUC than those of _log_hs-CRP (0.623 [0.543~0.699]), WBC (0.617 [0.536–0.693]), neutrophil (0.656 [0.577–0.730]), and lymphocyte (0.699 [0.621–0.769]). Lastly, the difference between _log_NLR and WBC and the difference between _log_NLR and neutrophil counts were statistically significant (Fig. 2b).

### Nomogram for predicting in-hospital death

**Fig. 3:**
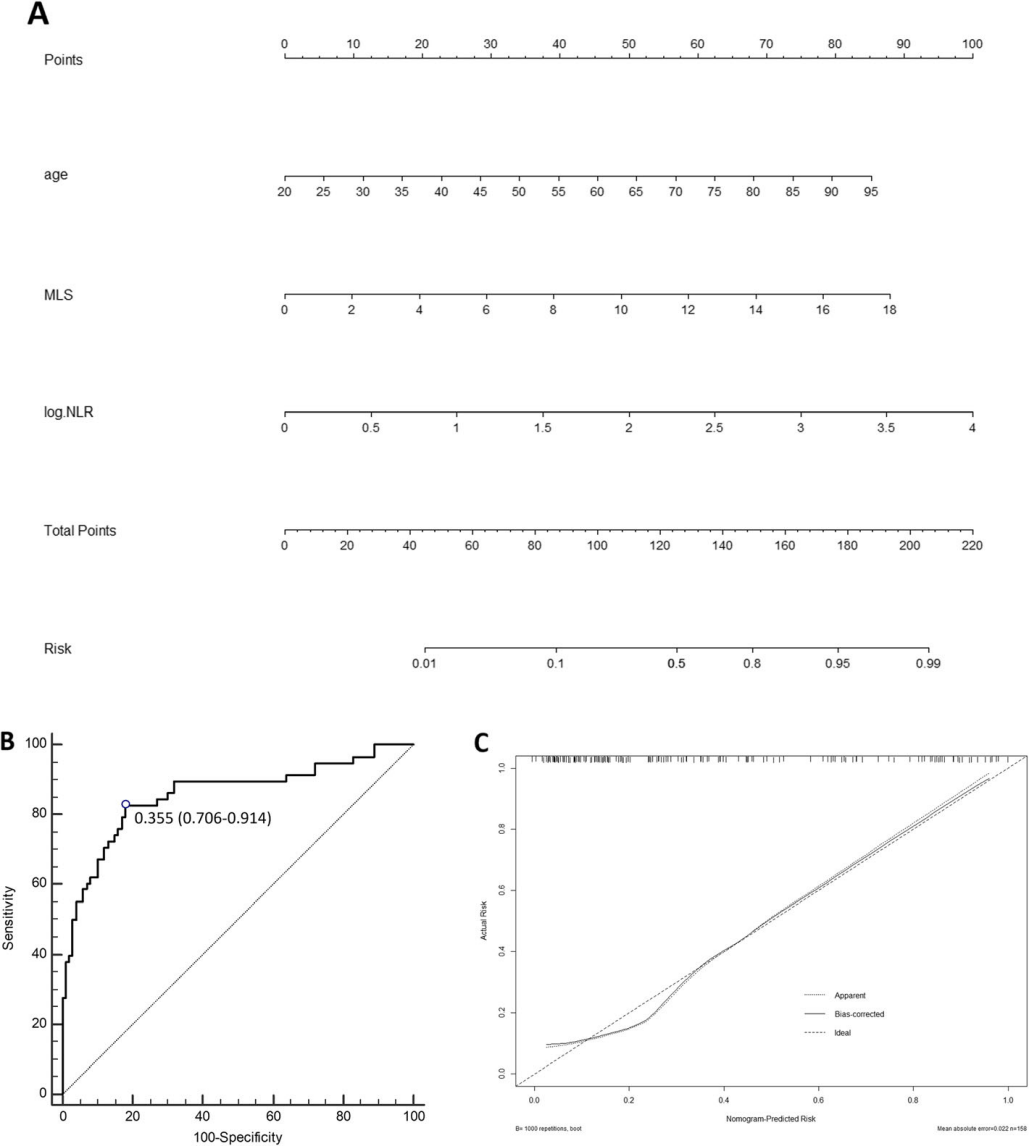
**a** Nomogram of the study population to predict in-hospital death in patients with LHI; **b**: ROC curve of the nomogram used for predicting in-hospital mortality in patients with LHI. The area under curve was 0.858 (95% CI, 0.794~0.908); **c**: Calibration curves for in-hospital mortality, which are representative of predictive accuracy. MLS: midline shift; NLR: neutrophil-to-lymphocyte ratio

Finally, we recruited all independent prognostic factors identified in multivariate logistic regression analysis of in-hospital death to construct nomograms (Fig. 3). Each variable is projected upward to the value of the small ruler (points) to get the score of each parameter. Summing the points assigned to the corresponding factors can get the total points. The higher the total score, the higher the risk of death. This nomogram can predict the in-hospital death individually according to the different conditions of different patients. We then assessed the predictive accuracy of this prognostic model. The AUC-ROC of this nomogram was 0.858 (95% CI, 0.794~0.908).

## Discussion

The high mortality is one of the most serious problems in patients with LHI, early identification of reliable predictors of in-hospital death should be a valuable perspective on precise clinical and care management. Reported predictors associated with poor prognosis of LHI patients included higher serum tissue inhibitor of matrix metalloproteinase-1 (TIMP-1) levels during the first week, rising serum nardilysin concentrations, intercaudate distance [16–18]. Favorable outcome depends on younger age, lower baseline NIHSS score, absence of brain edema and pneumonia and statins used in the acute phase [4]. In addition, previous clinical researches also indicated that pre-operation brain CT hypodensity volume, MCA with additional territory infarct, extent of preoperative midline shift, and postoperative consciousness level were associated with in-hospital mortality in patients with large hemispheric stroke who received palliative decompressive craniectomy [19, 20]. However, there are few studies focused on prognosis of patients with LHI who purely receives medical treatment. In this cohort, patients with large hemispheric infarction undergoing non-surgical management have a mortality of 36.7%, it’s much lower than previous studies (71% in previous randomized controlled trials of decompressive craniectomy) [6]. We suspect the reason is the younger age and the broader inclusion criteria. In this single-center retrospective study of LHI patients with conservative treatment, age, MLS and NLR values could independently predict in-hospital mortality.

Age is an acknowledged risk factor associated with poor prognosis after acute ischemic stroke, but the definitive relationship between age and hospital death is still in dispute. Previous studies on ischemic stroke included LHI patients suggested that younger age was associated with favorable prognosis in non-selective patients with ischemic stroke [4, 21–24]. However, for patients during hospitalization, it is generally agreed that development of space-occupying edema secondary to LHI led to further clinical deterioration and in-hospital death [1, 25]. Older patients may benefit from age-related brain atrophy, which could provide compensatory space for brain edema. However, at the same time, older patients with LHI are more likely to develop larger infarction volume, even the age-related brain atrophy was taken into account [26]. Recently, a meta-analysis (38 studies, 31 cohort studies and 7 case-control studies, in 49 articles) showed that younger age was an early predictor for malignant edema after stroke [27]. But in our cohort, age has positive correlation with in-hospital death (adjusted OR = 1.066, by each additional year, CI: 1.025~1.108), and we provide an optimal cutoff age of 60 years (63.0% sensitivity and 77.7% specificity). We speculate the higher fatality rate in older patients of our cohort is due to the fact that the older adults have increased susceptibility to more serious complication.

MLS is helpful in evaluating the severity of craniocerebral injury and the prognosis of patients, even before clinical symptoms worsen [28, 29]. Moreover, MLS is easy to obtain by CT or MRI, or bed-side transcranial sonography [28, 30, 31].. Furthermore, this study provides an optimal cut-off for in-hospital mortality of 5.4 mm (53.4% sensitivity and 87.0% specificity), not far different from previous studies [28, 32].

In the last few years, some studies have found that high NLR value correlates with early clinical outcomes in patients with acute ischemic stroke [10, 33, 34]. One possible explanation is the immunologic changes that occur after acute ischemic stroke. Immune responses and inflammation play a key role in the pathophysiology of ischemic stroke [35]. Acute ischemic stroke may obviously activate the immune system by reinforcing the migration of neutrophil in the brain, which increases the blood-brain barrier permeability [36]. Moreover, increased neutrophil releases MMP-9 (matrix metalloproteinase-9) and disrupts the blood-brain barrier, which may result in cerebral edema and hemorrhagic transformation after acute ischemic stroke (AIS) [34, 37]. Additionally, reduced lymphocyte count indicates that the protection on central nervous system mediated by regulatory B cells and regulatory T cells have weakened [35, 38]. These might account for the relationship between high NLR value and unfavorable prognosis in patients with acute ischemic stroke.

For patients with LHI, in-hospital deaths can be caused by many different factors. Currently, a 2-center retrospective cohort study (*N* = 1317) showed that high neutrophil-to-lymphocyte ratio can predict stroke-associated pneumonia (AUC = 0.876, [0.855~0.895]), which is one potential reason of patients’ worsening condition [39]. Patients with higher NLR values are thus likely to be subject to a variety of complications and consequently die in hospital. Finally, through comparisons of the predictive power of NLR and conventional inflammation markers, NLR is found to be a better predictor than WBC and neutrophil counts.

Patients with LHI showed a high mortality rate during hospitalization. While the sensitivity of three independent predictors in our cohort were not high enough (63.0% sensitivity of age, 53.4% sensitivity of MLS and 72.4% sensitivity of NLR), a reliable nomogram was established for the prediction of in-hospital death (AUC-ROC = 0.858, 95% CI, 0.794~0.908). Nomogram is widely used in oncology and medicine to predict the probability of clinical events by integrating different variables. With a straightforward digital interface, greater accuracy, and more easily understood prognoses, nomogram has the power to help clinicians make sound and timely decisions [40]. In recent years, it has also been applied in stroke prognosis [41, 42]. Furthermore, to the best of our knowledge, our study is the first to construct nomogram for the prediction of in-hospital death in patients with LHI.

Although LHI was associated with high mortality for a long time, early surgical treatment can reduce mortality and complications [43, 44]. Additionally, recent randomized controlled trial GAMES-RP indicated that intravenous glyburide also was effective against brain edema [45]. Meanwhile, anti-infection and preventing hemorrhagic transformation were other important measures to improve outcome of LHI patients. This visual nomogram can be used to aid clinicians make a correct decision.

### Limitation

There are several limitations in our study as follows. First of all, the database did not contain sufficient information of all patients. Some variables were not available to be taken into consideration, such as NIHSS scores. Therefore, the potential bias is inevitable. Second, as a single-center retrospective study, our data may not be fully representative. The results may need to be verified by other hospitals or other ethnic groups. Third, treatment details of individual patients varied but were not taken into account in this study, which might affect the prognosis of different patients. Moreover, we just have a limited number of cases, which may affect the credibility of the results. However, despite these limitations, we successfully identified three prognostic factors for patients with LHI after admission. More importantly, our study is the first to construct a nomogram model to predict the in-hospital mortality of LHI patients.

## Conclusion

In summary, the study shows that age, MLS, NLR values correlate with patients’ in-hospital mortality. NLR values should be considered in the management of LHI. An effective prediction model, nomogram is a promising tool for patients with ischemic stroke. This finding may be helpful for clinicians to suggest appropriate treatment for patients. Additionally, it is imperative to confirm these findings through prospective, multicenter, large-scale trials.

## Data Availability

The datasets used and analysed during the current study are available from the corresponding author on reasonable request.

## Abbreviations

AIS: Acute ischemic stroke
AUC: Area under the curve
CI: Confidence Interval
CT: Computed tomography
DBP: Baseline diastolic pressure
DHC: Decompressive hemicraniectomy
DWI: Diffusion weighted image
ECASS-II: European Cooperative Acute Stroke Study-II classification
GLU: Baseline blood glucose
HbA1c: Glycated hemoglobin
HDL: High-density lipoprotein
hs-CRP: high sensitivity C-reactive protein
IQR: Interquartile ranges
LDL: Low density lipoprotein
LHI: Large hemispheric infarction
MCA: Middle cerebral artery
MLS: Midline shift
MMI: Malignant middle cerebral artery infarction
MMP-9: Matrix metalloproteinase-9
MRI: Magnetic resonance imaging
mRS: Modified Rankin scale
NICU: Neuro-intensive care unit
NIHSS: National Institutes of Health Stroke Scale score
NLR: Neutrophil to lymphocyte ratio
OR: Odds Ratio
SBP: Baseline systolic pressure
SE: STANDARD Error
TG: Triglyceride
TIMP-1: Tissue inhibitor of matrix metalloproteinase-1
WBC: White blood count

## References

[1] Hacke W, Schwab S, Horn M, Spranger M, De Georgia M, von Kummer R (1996). ‘Malignant’ middle cerebral artery territory infarction: clinical course and prognostic signs. Arch Neurol.

[2] Huttner HB, Schwab S (2009). Malignant middle cerebral artery infarction: clinical characteristics, treatment strategies, and future perspectives. Lancet Neurol.

[3] Ong CJ, Gluckstein J, Laurido-Soto O, Yan Y, Dhar R, Lee JM (2017). Enhanced detection of edema in Malignant anterior circulation stroke (EDEMA) score: a risk prediction tool. Stroke..

[4] Li J, Zhang P, Wu S, Yi X, Wang C, Liu M (2018). Factors associated with favourable outcome in large hemispheric infarctions. BMC Neurol.

[5] Heinsius T, Bogousslavsky J, Van Melle G (1998). Large infarcts in the middle cerebral artery territory. Etiology and outcome patterns. Neurology..

[6] Vahedi K, Hofmeijer J, Juettler E, Vicaut E, George B, Algra A (2007). Early decompressive surgery in malignant infarction of the middle cerebral artery: a pooled analysis of three randomised controlled trials. Lancet Neurol.

[7] Juttler E, Unterberg A, Woitzik J, Bosel J, Amiri H, Sakowitz OW (2014). Hemicraniectomy in older patients with extensive middle-cerebral-artery stroke. N Engl J Med.

[8] Rahme R, Curry R, Kleindorfer D, Khoury JC, Ringer AJ, Kissela BM (2012). How often are patients with ischemic stroke eligible for decompressive hemicraniectomy?. Stroke..

[9] Pikija S, Sztriha LK, Killer-Oberpfalzer M, Weymayr F, Hecker C, Ramesmayer C (2018). Neutrophil to lymphocyte ratio predicts intracranial hemorrhage after endovascular thrombectomy in acute ischemic stroke. J Neuroinflammation.

[10] Brooks SD, Spears C, Cummings C, VanGilder RL, Stinehart KR, Gutmann L (2014). Admission neutrophil-lymphocyte ratio predicts 90 day outcome after endovascular stroke therapy. J Neurointerventional Surg.

[11] Heiss WD, Malignant MCA (2016). Infarction: Pathophysiology and Imaging for Early Diagnosis and Management Decisions. Cerebrovasc Dis.

[12] Uhl E, Kreth FW, Elias B, Goldammer A, Hempelmann RG, Liefner M (2004). Outcome and prognostic factors of hemicraniectomy for space occupying cerebral infarction. J Neurol Neurosurg Psychiatry.

[13] Sheth KN, Petersen NH, Cheung K, Elm JJ, Hinson HE, Molyneaux BJ (2018). Long-term outcomes in patients aged </=70 years with intravenous glyburide from the phase II GAMES-RP study of large hemispheric infarction: an exploratory analysis. Stroke..

[14] Donders AR, van der Heijden GJ, Stijnen T, Moons KG (2006). Review: a gentle introduction to imputation of missing values. J Clin Epidemiol.

[15] DeLong ER, DeLong DM, Clarke-Pearson DL (1988). Comparing the areas under two or more correlated receiver operating characteristic curves: a nonparametric approach. Biometrics..

[16] Lorente L, Martin MM, Ramos L, Argueso M, Caceres JJ, Sole-Violan J (2019). High serum levels of tissue inhibitor of matrix metalloproteinase-1 during the first week of a malignant middle cerebral artery infarction in non-surviving patients. BMC Neurol.

[17] Chen FH, Wang Y, Jiang YX, Zhang GH, Wang ZM, Yang H (2019). Clinical determination of serum nardilysin levels in predicting 30-day mortality among adults with malignant cerebral infarction. Clin Chim Acta.

[18] Beck C, Kruetzelmann A, Forkert ND, Juettler E, Singer OC, Kohrmann M (2014). A simple brain atrophy measure improves the prediction of malignant middle cerebral artery infarction by acute DWI lesion volume. J Neurol.

[19] Huang P, Lin FC, Su YF, Khor GT, Chen CH, Lin RT (2012). Predictors of in-hospital mortality and prognosis in patients with large hemispheric stroke receiving decompressive craniectomy. Br J Neurosurg.

[20] Kamran S, Salam A, Akhtar N, Alboudi A, Ahmad A, Khan R (2017). Predictors of in-hospital mortality after Decompressive Hemicraniectomy for Malignant ischemic stroke. J Stroke Cerebrovasc Dis.

[21] Mori K, Nakao Y, Yamamoto T, Maeda M (2004). Early external decompressive craniectomy with duroplasty improves functional recovery in patients with massive hemispheric embolic infarction: timing and indication of decompressive surgery for malignant cerebral infarction. Surg Neurol.

[22] Gupta R, Connolly ES, Mayer S, Elkind MS (2004). Hemicraniectomy for massive middle cerebral artery territory infarction: a systematic review. Stroke..

[23] Nakayama H, Jorgensen HS, Raaschou HO, Olsen TS (1994). The influence of age on stroke outcome. Copenhagen Stroke Stud Stroke.

[24] Macciocchi SN, Diamond PT, Alves WM, Mertz T (1998). Ischemic stroke: relation of age, lesion location, and initial neurologic deficit to functional outcome. Arch Phys Med Rehabil.

[25] Zhao J, Su YY, Zhang Y, Zhang YZ, Zhao R, Wang L (2012). Decompressive hemicraniectomy in malignant middle cerebral artery infarct: a randomized controlled trial enrolling patients up to 80 years old. Neurocrit Care.

[26] Goto Y, Kumura E, Watabe T, Nakamura H, Nishino A, Koyama T (2016). Prediction of Malignant middle cerebral artery infarction in elderly patients. J Stroke Cerebrovasc Dis.

[27] Wu S, Yuan R, Wang Y, Wei C, Zhang S, Yang X (2018). Early prediction of Malignant brain edema after ischemic stroke. Stroke..

[28] Gerriets T, Stolz E, Konig S, Babacan S, Fiss I, Jauss M (2001). Sonographic monitoring of midline shift in space-occupying stroke: an early outcome predictor. Stroke..

[29] Walberer M, Blaes F, Stolz E, Muller C, Schoenburg M, Tschernatsch M (2007). Midline-shift corresponds to the amount of brain edema early after hemispheric stroke--an MRI study in rats. J Neurosurg Anesthesiol.

[30] Stolz E, Gerriets T, Fiss I, Babacan SS, Seidel G, Kaps M (1999). Comparison of transcranial color-coded duplex sonography and cranial CT measurements for determining third ventricle midline shift in space-occupying stroke. AJNR Am J Neuroradiol.

[31] Gerriets T, Stolz E, Modrau B, Fiss I, Seidel G, Kaps M (1999). Sonographic monitoring of midline shift in hemispheric infarctions. Neurology..

[32] Park J, Goh DH, Sung JK, Hwang YH, Kang DH, Kim Y (2012). Timely assessment of infarct volume and brain atrophy in acute hemispheric infarction for early surgical decompression: strict cutoff criteria with high specificity. Acta Neurochir.

[33] Yu S, Arima H, Bertmar C, Clarke S, Herkes G, Krause M (2018). Neutrophil to lymphocyte ratio and early clinical outcomes in patients with acute ischemic stroke. J Neurol Sci.

[34] Qun S, Tang Y, Sun J, Liu Z, Wu J, Zhang J (2017). Neutrophil-to-lymphocyte ratio predicts 3-month outcome of acute ischemic stroke. Neurotox Res.

[35] Lakhan SE, Kirchgessner A, Hofer M (2009). Inflammatory mechanisms in ischemic stroke: therapeutic approaches. J Transl Med.

[36] Jickling GC, Liu D, Stamova B, Ander BP, Zhan X, Lu A (2014). Hemorrhagic transformation after ischemic stroke in animals and humans. J Cerebral Blood Flow Metab.

[37] Montaner J, Molina CA, Monasterio J, Abilleira S, Arenillas JF, Ribo M (2003). Matrix metalloproteinase-9 pretreatment level predicts intracranial hemorrhagic complications after thrombolysis in human stroke. Circulation..

[38] Ren X, Akiyoshi K, Dziennis S, Vandenbark AA, Herson PS, Hurn PD (2011). Regulatory B cells limit CNS inflammation and neurologic deficits in murine experimental stroke. J Neurosci.

[39] Nam KW, Kim TJ, Lee JS, Kwon HM, Lee YS, Ko SB (2018). High neutrophil-to-lymphocyte ratio predicts stroke-associated pneumonia. Stroke..

[40] Balachandran VP, Gonen M, Smith JJ, DeMatteo RP (2015). Nomograms in oncology: more than meets the eye. Lancet Oncol.

[41] Deng QW, Li S, Wang H, Lei L, Zhang HQ, Gu ZT (2018). The short-term prognostic value of the triglyceride-to-high-density lipoprotein cholesterol ratio in acute ischemic stroke. Aging Dis.

[42] Turcato G, Cervellin G, Cappellari M, Bonora A, Zannoni M, Bovi P (2017). Early function decline after ischemic stroke can be predicted by a nomogram based on age, use of thrombolysis, RDW and NIHSS score at admission. J Thromb Thrombolysis.

[43] Cho DY, Chen TC, Lee HC (2003). Ultra-early decompressive craniectomy for malignant middle cerebral artery infarction. Surg Neurol.

[44] Waziri A, Fusco D, Mayer SA, McKhann GM, Connolly ES (2007). Postoperative hydrocephalus in patients undergoing decompressive hemicraniectomy for ischemic or hemorrhagic stroke. Neurosurgery..

[45] Sheth KN, Elm JJ, Molyneaux BJ, Hinson H, Beslow LA, Sze GK (2016). Safety and efficacy of intravenous glyburide on brain swelling after large hemispheric infarction (GAMES-RP): a randomised, double-blind, placebo-controlled phase 2 trial. Lancet Neurol.

